# Potential impact of a nonavalent anti HPV vaccine in Italian men with and without clinical manifestations

**DOI:** 10.1038/s41598-021-83639-6

**Published:** 2021-02-18

**Authors:** Liana Bosco, Nicola Serra, Teresa Fasciana, Daniela Pistoia, Marco Vella, Leonardo Di Gregorio, Rosaria Schillaci, Antonino Perino, Gloria Calagna, Alberto Firenze, Giuseppina Capra

**Affiliations:** 1 Department of Biomedicine, Neuroscience and Advanced Diagnostics (Bi.N.D.), Section of Biology and Genetics, University of Palermo, Palermo, Italy; 2 Department of Molecular Medicine and Medical Biotechnology, University Federico II of Naples, Naples, Italy; 3 Department of Health Promotion, Mother and Child Care, Internal Medicine and Medical Specialties (ProMISE) “G. D’Alessandro”, University of Palermo, Via del Vespro, 133, 90127 Palermo, Italy; 4 UOC of Microbiology, Virology and Parasitology, Polyclinic Hospital, Palermo, Italy; 5 Section of Urology, Department of Surgical, Oncological and Oral Sciences, University of Palermo, Palermo, Italy; 6 UOC of Urology and Extracorporeal Lithotripsy, Polyclinic Hospital, Palermo, Italy; 7 Villa Sofia Cervello Hospital, University of Palermo, Palermo, Italy

**Keywords:** Microbiology, Diseases, Health care, Medical research

## Abstract

Human papilloma virus infection (HPV) is the most common sexually transmitted disease. Little is known about male infection. Nonavalent vaccine against types 6/11/16/18/31/33/45/52/58 was approved and neutral gender immunization programs have been proposed. This study evaluates the potential impact of nonavalent vaccine compared to quadrivalent in male living in Sicily (Italy). 58.7% of samples were HPV positive and forty-four types of HPV were identified. A significant higher estimated coverage of nonavalent vaccine than quadrivalent was observed (64.3% vs. 45.8%), with absolute and relative additional impact of 20.1% and 47.2%, respectively. Low impact of the vaccine were calculated as the empirical probability of HPV genotypes 6/11/16/18/31/33/45/52/58 alone or in combination; the high impact as empirical probability of HPV6/11/16/18/31/33/45/52/58 genotypes alone or in association with other genotypes. The potential impact of the nonavalent vaccine *vs* quadrivalent was significant for low and high impact (29.7% > 18:8%; 34:6% > 26.6%, respectively). Particularly, in men with lesions and risky sexual contact was significant only for low impact (35.5% > 29.7%; 31.4% > 19.7%, respectively). In partners with positive females was significant for low impact (26.3% > 15.1%) and high impact (33.7% > 23.2%). Nonavalent vaccine offers broader protection in men with HPV positive partners, who would have a potential role in the transmission of the infection.

## Introduction

Human PapillomaVirus (HPV) infection is the most common sexually transmitted disease in the world^1^. There are more than 200 different types of human papillomaviruses, and around 40 that affect the anogenital area^2^. Most of the time HPV infections are asymptomatic, transient and disappear without treatment in a short time. Some infections, however, can persist for many years, causing cellular changes that, if left untreated, have the potential to develop into cancer of the anogenital area (carcinoma of the cervix, vagina, vulva, anal, oral and penis)^3–8^ or in benign proliferative lesions, warts and anogenital papillomas, oral papillomas, and forms of recurrent respiratory papillomatosis in men and women^9–12^. In contrast to the wealth of information available on the rate of and risk factors for acquisition of HPV infection in women, much less is known about such factors in men. There is great variation in the prevalence depending on anatomical sites sampled, sampling methods, and HPV DNA detection assays^13–15^. HPV infection prevalence varies greatly in the men population and reaches 65.4% between 18 and 40 years^16^. Researchers have documented that 50–77% of steady male partners of women with HPV infection, cervical neoplasia, or both have subclinical HPV infection^17,18^, in addition to being an indirect causal factor for cervical cancer via men serving as reservoirs for HPV transmission. Therefore, monitoring for the presence of various HPV genotypes might be important, especially in those patients with known high risk HPV (hrHPV) type^19^.

To prevent HPV infection, a widespread immunization campaign has been approved in adolescent females around the world. More recently, the implementation of vaccination has been recommended in boys and young men as well^20^ and a universal immunization program has been proposed^21,22^. The quadrivalent vaccine (GARDASIL, MERCK & CO., Inc., Whitehouse Station, NJ) protects against 4 HPV types: 6, 11, 16, and 18. Gardasil prevents persistent infections and genital diseases caused by these HPV types in females. Moreover, it has been demonstrated a protective effect of quadrivalent among heterosexual males (92% efficacy) and its use was licensed in young males to prevent anogenital condyloma. In 2014 the U.S. Food and Drug Administration has approved the nonavalent vaccine (GARDASIL, MERCK & CO., Inc., Whitehouse Station, NJ). It is a vaccine given to individuals 9 through 45 years of age to help protect against diseases caused by HPV types 6, 11, 16, 18, 31, 33, 45, 52, 58. Since 2018 all EU/EEA countries has introduced HPV vaccination in their national immunization programs^23^.

The switch from a first generation HPV vaccines to a nonavalent vaccine increases the prevention of high grade cervical lesions up to 90% of cases^24^. Capra et al. showed, by high estimate calculations, that the absolute additional impact of the nonavalent HPV vaccine is substantial for both low-grade squamous intraepithelial lesion (LSIL) and high-grade squamous intraepithelial lesion (HSIL), with a 23.8–32.8% increase in the proportion of cases effectively prevented by the second generation HPV vaccine (absolute additional impact)^25^.

The aim of this study was to assess HPV genotype distribution among men with and without clinical manifestations, and to evaluate the potential impact of the nonavalent HPV vaccine on HPV infection compared with the previously utilized quadrivalent HPV vaccine, in a male population living in Sicily, Southern Italy.

## Materials and methods

### Study population

The subject of this study is the male population living in Sicily, Southern Italy. The analysis involved 975 consecutive men who had come to the Virology laboratory at the of the Department of Health Promotion, Mother and Child Care, Internal Medicine and Medical Specialties-ProMISE (University of Palermo, Polyclinic Hospital, Italy) between January 2015 and December 2019, with a request for HPV testing.The common reasons for requesting an HPV test included men with clinical lesions (genital or anal warts, atypical penile lesions); men having a risky sexual contact in the prior 2 months (sexual transmitted diseases (STD) evaluation);men having an HPV positive partner.

A visual inspection (hand-held lens) was carried out to assess the presence of clinical symptoms such as atypical lesions or warts on the external genitals. After HPV testing and evaluation of clinical symptoms three series of genital samples were selected and examined as follows:

- Group I: 200 genital samples of men with presence of clinical symptoms;
- Group II: 309 genital samples of men with a risky sexual contact;
- Group III: 466 genital samples of men with HPV positive partner.

### Inclusion and exclusion criteria

All patients who met the following inclusion criteria were enrolled: men with age between 18 and 77 years; men with clinical symptoms; men with risky sexual contact; men positive partner for hrHPV. The exclusion criteria were: varicocele; cryptorchidism; other genital infections; patients treated with different medical therapies (chemo/radiotherapy), patients with clinical characteristics that did not allow exclusive inclusion in a single study group. These patients were excluded from the study.

### Ethical statement

All study subjects provided written informed consent. Study participants authorized the research responsible to use biological specimens collected for scientific purposes. They also expressed their agreement to participate to medical and sexual interviews. The research was conducted in accordance with the Helsinki Declaration; the informed consent and the study protocol were approved by the Institutional Review Board at the Policlinico, University of Palermo (Italy). The study did not provide financial compensation for the participants. The authors are grateful to the host institution:University Hospital Polyclinic, who made the fund available PSN2018-4.23, entitled "Vaginal microbiota in physiological and pathological conditions with particular reference to HPV infections". 

### Genital sampling

Samples were collected from penis and urethra. For HPV sampling, patients were instructed to avoid washing their genitalia the day before the examination and to observe 2 days of sexual abstinence before the examination. Genital sampling was performed as described previously^13^, with some modifications. In brief, for penile brushing, cells from the dorsal/ventral surface of the shaft were collected by a standard-sized, dry DACRON swab first, and then by a saline-wetted Dacron swab. Cells from the inner foreskin, coronal sulcus, frenulum and glans were also collected using a saline-wetted cytobrush. Five to six back-and-forth movements of the swab/cytobrush were performed at each penile site. For urethral sampling, a very thin, saline-wetted cytobrush was inserted 1.5 cm into the urethra, rotated 360 degrees, and removed. The urethral cells were placed in a separate vial with 3 ml of phosphate-buffered saline. The samples were processed for DNA extraction immediately following collection.

### DNA extraction and HPV testing

Cells obtained from penile and urethral brushing were spun down at 13,000 rpm for 5 min and total DNA was extracted with the use of the High Pure PCR Template Preparation kit (ROCHE DIAGNOSTICS GMBH, Mannheim, Germany), following manufacturer’s instructions. DNA quality and the absence of inhibitors were confirmed by testing for the human β-globin gene, as described elsewhere. Amplifications were carried out in a DNA thermal cycler (Mastercycler, EPPENDORF, Hamburg, Germany) and the PCR products were analyzed in 8% polyacrylamide gel^26^.

The presence of HPV DNA was detected using two HPV assays. The INNO-LiPA HPV Genotyping Extra II kit (FUJIREBIO DIAGNOSTICS, INC, Great Valley Parkway, Malven), based on the combined use of SPF10 PCR and LiPA hybridization, was employed. The SPF general primers detect at least 43 different HPV genotypes^27^ and the LiPA type-specific assay identifies thirty-two types. 20 hrHPV (HPV16, HPV18, HPV26, HPV31, HPV33, HPV35, HPV39, HPV45, HPV51, HPV52, HPV53, HPV56, HPV58, HPV59, HPV68, HPV66, HPV67, HPV70, HPV73 and HPV82), 12 low-risk HPV (lrHPV): HPV6, HPV11, HPV40, HPV42, HPV43, HPV44, HPV54, HPV61, HPV62 HPV81, HPV83 and HPV89). Due to the higher number of HPV types detected by the SPF10 primers compared to the LiPA assay, some samples yielded SPF10-positive/LiPA negative results. These HPV types were subsequently amplified by a highly sensitive nested PCR assay, consisting of a first step of amplification with the PGMY09/11 primer pair, followed by the second step with the GP05 + /GP06 + primers, as previously described^26,28^. The HPV genotyping procedure was based on the direct sequencing of GP-PCR fragments, utilizing consensus nested primers as sequencing primers, as described elsewhere^29^.

### Sample size estimation

To individualize a sample sizes statistically significant for this study, we considered a Bernoulli sampling^30^.

The minimum sample size was estimated equal to 350 patients HPV-positive. It was obtained considering a statistical z-score at 95%, an error ε = 5% and hypothesizing a prevalence π of 35.4%, obtained from penile and urethral samples, including patients with presence of clinical symptoms,with a risky sexual contact, and with HPV positive partner, according to Barzon, in this way we estimated a prevalence range equal to 30.4–40.4%^31^.

Consequently, the minimum sample size for each group considered in this study was estimated equal to 117 patients. Finally, the sample size for each group was enlarged considering the possibility of unexpected events and consequently the possibility of patients data loss, and to minimize possible statistical biases. Therefore, we consider Group I, Group II and Group III composed by 138, 137 and 297 patients HPV-positive.

### Statistical analysis

Data are presented as number and percentage for categorical variables, and continuous data expressed as the mean ± standard deviation (SD) unless otherwise specified. In order to compare the impact of nonavalent HPV vaccine with the quadrivalent vaccine we used the following statistical tests.

The χ^2^ test was performed to test the differences between two unpaired proportions or percentages. The McNemar’s exact test was used to test the difference between two paired proportions or percentages.

Multiple chi-square test was used to define significant differences among independent groups. In this case, if the chi-square test was significant (p < 0.05), a post hoc Z-test was performed to identify the highest or lowest significant frequency. In addition tests on continuous data were performed with one-way ANOVA test to evaluate significant differences among independent groups. If the ANOVA test was positive, post hoc test with Scheffé's method was performed for pairwise comparison of subgroups.

About vaccine impact, we used similar parameters adopted by Riethmuller et al.^24^, particularly, we considered the empirical probability evaluated on positive cases only. In this way, we define *low impact*, as the empirical probability of HPV genotypes (HPV6,11,16 and 18 for the quadrivalent, and HPV6,11,16,18,31,33,45,52 and 58 for the nonavalent vaccine), alone or in association, by excluding the presence of any other HPV type; while with *high impact*, the empirical probability of HPV genotypes (HPV6,11,16 and 18 for the quadrivalent, and HPV6,11,16,18,31,33,45,52 and 58 for the nonavalent vaccine) alone or in association, also in the presence of any other HPV type. These parameters have been defined in order to be able to optimally compare the effectiveness of the two vaccines.. Furthermore, sample size estimation was performed to ensure an adequate group size to obtain a statistically significant and robust study.

In addition, we computed both absolute and relative additional potential impact of the nonavalent vaccine compared to the quadrivalent vaccine. Particularly the absolute additional potential impact of the nonavalent vaccine, i.e., the proportion of additional cases potentially prevented by the nonavalent vaccine compared to the quadrivalent vaccine, was calculated as [(*n*_nonavalent_-*n*_quadrivalent_)/*N*] × 100, with *n* being the number of lesions/infections potentially prevented and *N* the total number of lesions/infections. Instead, the relative additional potential impact of the nonavalent vaccine compared to the quadrivalent vaccine was calculated as [(*n*_nonavalent_-*n*_quadrivalent_)/*n*_quadrivalent_] × 100 with *n* representing the number of potentially prevented lesions/infections.^24^.

Finally, all tests with p-value (p) < 0.05 were considered significant. The statistical analysis was performed using the Matrix Laboratory (MATLAB) analytical toolbox version 2008 (MATHWORKS, Natick, MA, USA). for Windows at 32 bit.

## Results

Of 975 genital samples β-globin-positive 572 (58.7%) samples tested were HPV positive, characterized by mean ± SD age equal to 36.0 ± 10.11 (range 19–77 years).

In Table 1, we show the general characteristics of 572 positive HPV patients, while in Table 2 we show the characteristics the HPV positive patients grouped in three categories. In this case we had HPV positive patients in 69% (138/200) in Group I, 44.3% (137/309) in Group II and 63.7% (297/466) in Group III. Particularly the last column of Table 2, we performed the statistical analysis among groups for each parameter considered. We observed by multivariate analysis, a significant relationship between groups and age (p = 0.015), and in particular the Group II was that with patients with age significant more high in comparison to Group I and III (38.61% vs. 35.38%, p < 0.05; 38.61% vs. 36.04%, p < 0.05; respectively).

**Table 1:** General characteristics of 572 positive HPV patients.

Parameters	Percentages/mean ± SD
HPV +	58.7% (572/975)
HPV−	41.3% (403/975)
HR-HPV	79.4% (454/572)
LR-HPV	20.6% (118/572)
**Category**	
Warts/Atypical lesion	24.1% (138/572)
Evaluation STD	23.9% (137/572)
Positive partner	51.9% (297/572)
Total single HPV	48.8% (279/572)
Total multiple HPV	51.2% (293/572)
2 HPV genotype	45% (132/293)
3 HPV genotype	24.9% (73/293)
4 HPV genotype	17.7% (52/293)
5 HPV genotype	7.2% (21/293)
6 HPV genotype	2.4% (7/293)
7 HPV genotype	1.4% (4/293)
8 HPV genotype	1% (3/293)
9 HPV genotype	0.3% (1/293)
**Vaccine quadrivalent genotype**	
Low impact	19.8% (113/572)
High impact	26.6% (152/572)
**Vaccine nonavalent genotype**	
Low impact	29.7% (192/572)
High impact	34.6% (198/572)
% AAI	20.1% (390–265 = 125/572)
% RAI	47.2% (390–265 = 125/265)

*LR* low risk, *HR* high risk, *AAI* absolute additional impact, *RAI* relative additional impact.

**Table 2:** Characteristics of pathologic patients grouped for disease.

Parameters	Group I 200 men with presence of clinical symptoms	Group II 309 men with a risky sexual contact (STD)	Group III 466 men with HPV positive partner	Statistical analysis among groups
HPV +	69% (138/200)	44.3% (137/309)	63.7% (297/466)	p < 0.0001* (C) Group I**, p = 0.0098 (Z) Group II***, p < 0.0001 (Z)
Age (HPV +)	35.38 ± 10.40	38.61 ± 10.79	36.04 ± 9.50	p = 0.015* (A) Group II > Group I, p < 0.05* (S) Group II > Group III, p < 0.05* (S)
HR-HPV	69.6% (96/138)	78.1% (107/137)	84.5% (251/297)	p = 0.0015* (C) Group I***, p = 0.0169 (Z)
LR-HPV	30.43% (42/138)	21.90% (30/137)	15.49% (46/297)	p = 0.0015* (C) Group I**, p = 0.0169 (Z)
Total single HPV	54.3% (75/138)	46.7% (64/137)	47.1% (140/297)	p = 0.322 (C)
Total multiple HPV	45.6% (63/138)	53.3% (73/137)	52.9 (157/297)	p = 0.322 (C)
2 HPV genotype	50.8% (32/63)	35.6% (26/73)	47.1% (74/157)	p = 0.154 (C)
3 HPV genotype	19% (12/63)	26% (19/73)	26.7% (42/157)	p = 0.475 (C)
4 HPV genotype	12.7% (8/63)	26.% (19/73)	15.9% (25/157)	p = 0.087 (C)
5 HPV genotype	7.9% (5/63)	6.8% (5/73)	7% (11/157)	p = 0.964 (C)
6 HPV genotype	3.2% (2/63)	2.7% (2/73)	1.9% (3/157)	p = 0.836 (C)
7 HPV genotype	4.8% (3/63)	0.00% (0/73)	1.3% (2/157)	p = 0.084 (C)
8 HPV genotype	1.6% (1/63)	1.4% (1/73)	0.00% (0/157)	p = 0.309 (C)
9 HPV genotype	0.00% (0/63)	1.4% (1/73)	0.00% (0/157)	p = 0.221 (C)
**Vaccine impact quadrivalent genotype**				
Low impact	29.7% (41/138)	19.7% (27/137)	15.1% (45/297)	p = 0.0018* (C) Group I**, p = 0.0137 (Z)
High impact	28.3% (39/138)	31.4% (43/137)	23.2% (69/297)	p = 0.171 (C)
**Vaccine impact nonavalent genotype**				
Low impact	35.5% (49/138)	31.4% (43/137)	26.3% (78/297)	p = 0.129 (C)
High impact	32.1% (45/138)	38% (52/137)	33.7% (100/297)	p = 0.60 (C)
% AAI	10.1% (14/138)	18.2% (25/137)	21.5% (64/297)	p = 0.0157* (C) Group I***, p = 0.0458 (Z)
% RAI	17.5% (14/80)	35.7% (25/70)	56.1% (64/114)	p < 0.0001* (C) Group III**, p = 0.0059 (Z) Group I***, p = 0.0007 (Z)

*AAI* absolute additional impact, *RAI* relative additional impact, *clinical symptoms* condiloma, atypical lesions.

*Significant test, **most frequent, ***less frequent; C = multiple comparison χ^2^ test; Z = Z-test; A = one way Anova test; S = Scheffé test for pairwise comparisons, post hoc Anova test.

**Figure 1:**
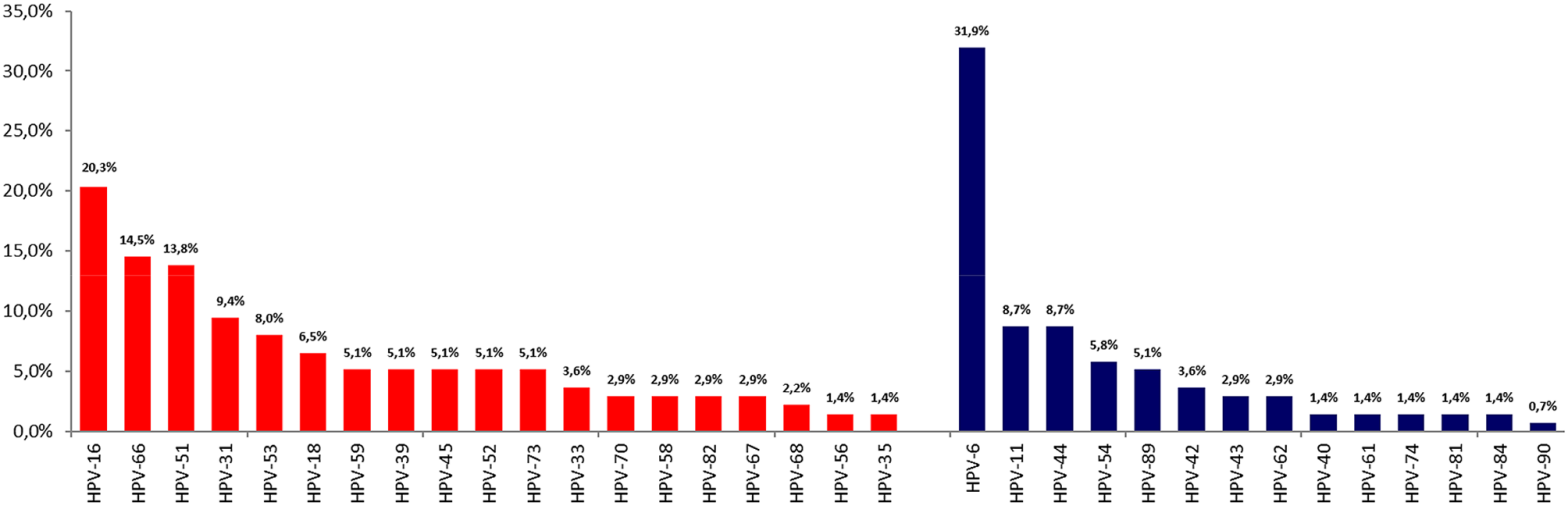
Type-specific distribution of hrHPV (red) and lrHPV (blue) among 138 men HPV + with presence of clinical lesions (Group I).

**Figure 2:**
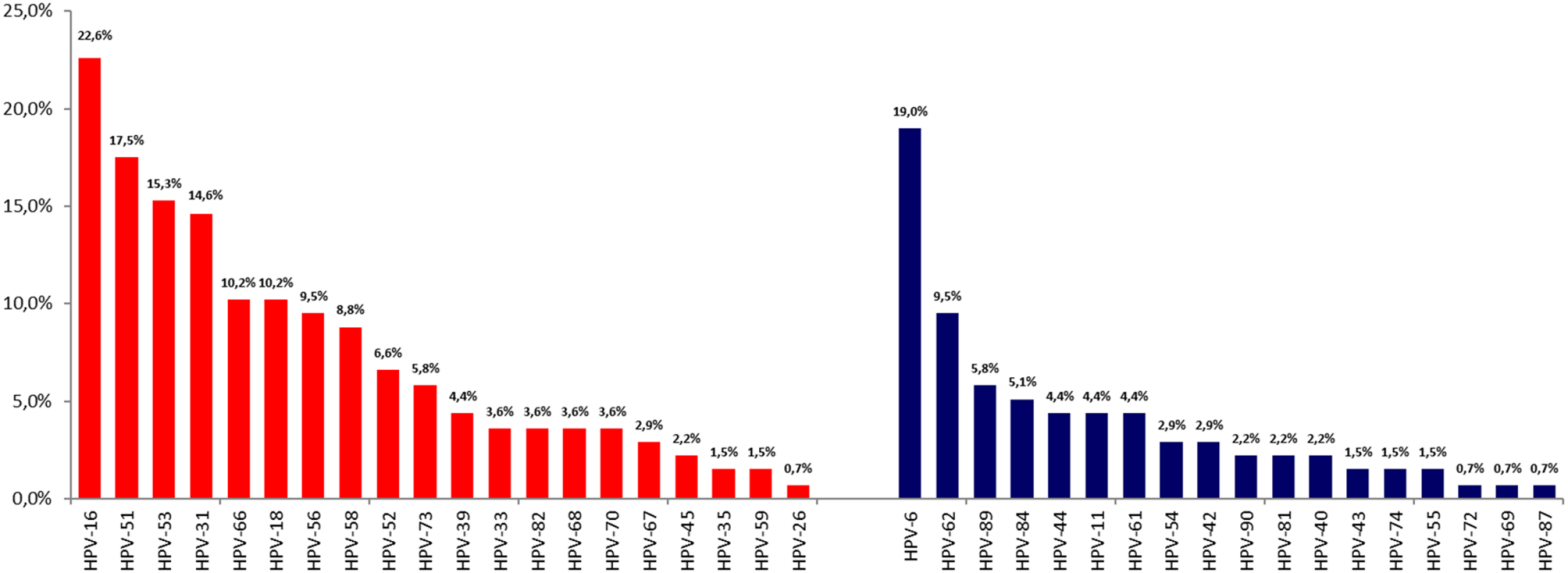
Type-specific distribution of hrHPV (red) and lrHPV (blue) among 137 men HPV + with a risky sexual contact (Group II).

**Figure 3:**
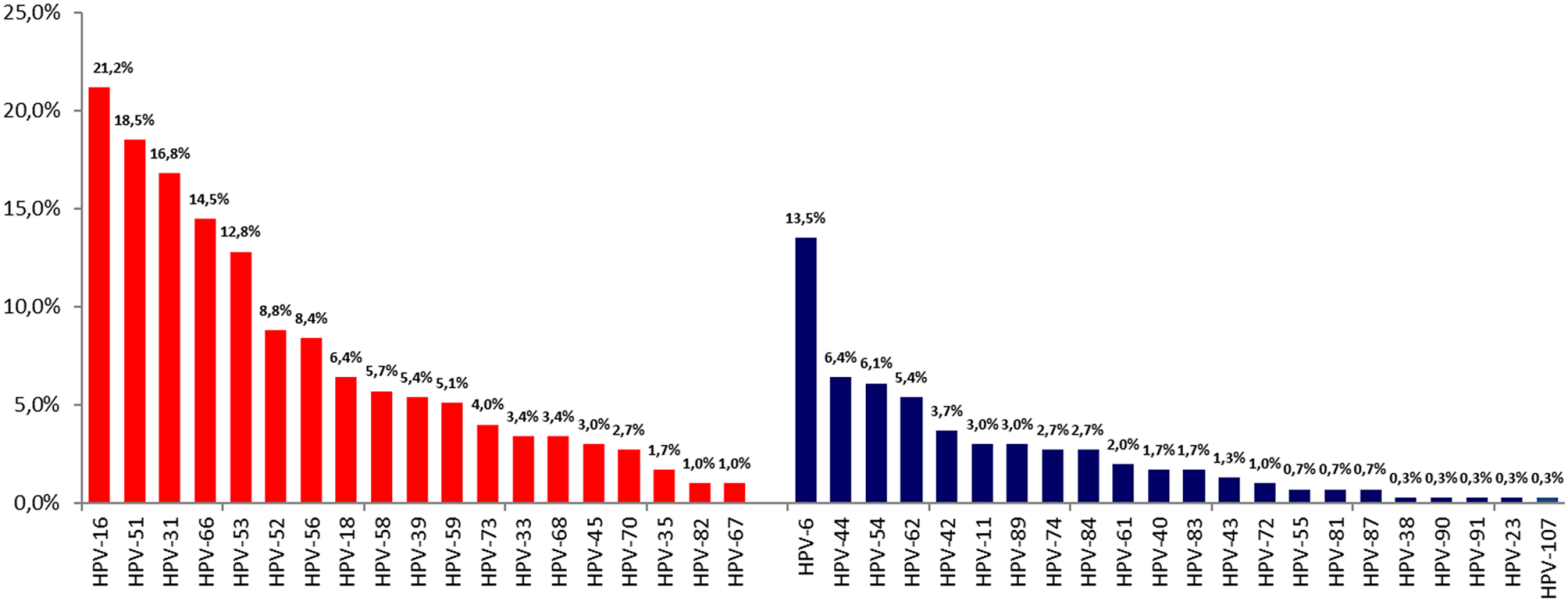
Type-specific distribution of hihrHPV (red) and lrHPV (blue) among 297 men HPV + with HPV positive partner (Group III).

Forty-four different HPV types were identified, of which 20 were hrHPV types and 24 lrHPV. The genotype most frequently identified was HPV6 (31.9% of HPV positive patients), HPV16 (20.3%) and HPV66 (14.5%) in Group I; HPV16 (22.6%) HPV51 (17.5%) and HPV53 (15.3%) in Group II, and HPV16 (21.2%), HPV51 (18.5%) and HPV31 (16.8%) in Group III. The percentage of identified genotypes in the three groups were reported in Figs. 1, 2 and 3.

As expected, about hrHPV, we found a significant relationship with groups (p = 0.0015). Particularly, in Group I was significant less frequent (69.6%, p = 0.0169) and lrHPV was significant more frequent (30.43%, p = 0.0169).

Multiple HPV type infections were shown in 51.2% (293/572) of the samples: 45.6% (63/138) of the Group I, 53.3% (73/137) of the Group II, and 52.9% (157/297) of the Group III, respectively. Oncogenic types were found in 79.4% (454/572); in particular, in 69.6% (96/138) of the Group I, in 78.1% (107/137) of the Group II and in 84.5% (251/297) in the Group III. Prevalence of oncogenic types, and the distribution of single and multiple infections in the different categories of men are shown in Tables 1 and 2.

From Table 3, 45.8% (262/572) of the genital samples harboured at least one of the four HPV types covered by the quadrivalent vaccine (HPV 6, 11, 16 and 18), while the 64.3% (368/572) of the samples harboured at least one of the nine genotypes included in nonavalent vaccine (HPV 6, 11, 16, 18, 31, 33, 45, 52 and 58), implying a significantly higher estimated coverage of HPV infection from the nonavalent vaccine than the current quadrivalent vaccine (64.3% vs. 45.8%; p < 0.0001).

**Table 3:** Percentages of patients with genotype included in quadrivalent and nonavalent vaccine.

Group	% patients with genotype included in quadrivalentvaccine	% patients with genotype included in nonavalent vaccine	Quadrivalent vs. nonavalent p-value (p)
Group I	58% (80/138)	67.4% (93/138)	58% < 67.4%, p = 0.0002* (M)
Group II	50.4% (69/137)	70.1% (96/137)	50.4% < 70.1%, p < 0.0001* (M)
Group III	38.% (113/297)	59.6% (177/297)	38% < 59.6%, p < 0.0001* (M)
Statistical analysis among groups++	p = 0.0003* (C) Group I**, p = 0.0153 (Z)	p = 0.068 (C)	
Total group	45.8% (262/572)	64.3% (368/572)	45.8% < 64.3%, p < 0.0001* (M)

*Significant test, **Most frequent, ***Less frequent; C = multiple comparison χ^2^ test; Z = Z-test; M = McNemar exact test; ++  = the statistical analysis was performed for column; p = p-value.

In addition, for low impact, Absolute Additional Impact (AAI), and Relative Additional Impact (RAI) a significant relationship with groups was found (p = 0.0018, p = 0.0157 and p < 0.001, respectively). Particularly, the Group I was significant more frequent for low impact (29.7%, p = 0.0137), while was less frequent for AAI and RAI (10.1%, p = 0.0458; 17.5%, p = 0.0007 respectively). Finally, the Group III was more frequent only for RAI (56.1%, p = 0.0059), as reported in Table 2**.**

In Table 3 we reported, a significant relationship between vaccine quadrivalent and group was found (p = 0.0003), particularly by post hoc Z-test, in Group I there was more present of patients in comparison to other groups (58%, p = 0.0153); while no significant relationship nonavalent vaccine and group was found (67.4%, 70.1%, 59.6%, p = 0.068). In addition, nonavalent vaccine vs quadrivalent vaccine implying a significantly higher estimated coverage for each group (Group I: 67.4% vs. 58%, p = 0.0002; Group II: 70.1% vs. 50.4%, p < 0.0001; Group III: 59.6% *vs.* 38.1%, p < 0.0001).

Finally, Table 4 shows the potential impact of quadrivalent and nonavalent HPV vaccines, as assessed by low and high impact estimates on the total of the samples and for each group.

**Table 4:** Comparison between vaccine nonavalent and quadrivalent, considering all positive HPV patients and all subgroups defined in this study.

	Vaccine quadrivalent genotype	Vaccine nonavalent genotype	Quadrivalent vs. Nonavalent p-value (p)
**All HPV positive patients (572)**			
Vaccine impact			
Low Impact	19.8% (113/572)	29.7% (170/572)	19.8% < 29.7%, p* < 0.0001 (M)
High Impact	26.6% (152/572)	34.6% (198/572)	26.6% < 34.6%, p* < 0.0001 (M)
**Group I** **138 positive HPV patients**			
Vaccine impact			
Low Impact	29.7% (41/138)	35.5% (49/138)	29.7% < 35.5%, p* = 0.0078 (M)
High Impact	28.3% (39/138)	32.6% (45/138)	28.3% < 32.6%, p = 0.146 (M)
**Group II** **137 positive HPV patients**			
Vaccine impact			
Low Impact	19.7% (27/137)	31.4% (43/137)	19.7% < 31.4%, p* < 0.0001 (M)
High Impact	31.4% (43/137)	38% (52/137)	31.4% < 38%, p = 0.0931 (M)
**Group III** **297 positive HPV patients**			
Vaccine impact			
Low Impact	15.1% (45/297)	26.3% (78/297)	15.1% < 26.3%, p* < 0.0001 (M)
High Impact	23.2% (69/297)	33.7% (100/297)	23.2% < 33.7%, p* < 0.0001 (M)

*Significant test; *M* McNemar exact test.

On the whole sample of 572 positive males, the potential impact of the quadrivalent and nonavalent vaccine varied significantly for the low impact between 19.8% and 29.7% (p < 0.0001) and for high impact between 26.6% and34.6% (p < 0.0001).

With regards to the sample of men with lesions and with STD evaluation (Group I and II), the potential impact of the nonavalent vaccine *vs* the quadrivalent vaccine was only effective for the low impact 35.5% > 29.7 (p = 0.0078) for Group I; and 31.4% > 19.7% (p < 0.0001) for Group II. In the first Group the absolute and relative additional impact of the nonavalent *vs* quadrivalent vaccine was 10.1% and 17.5%; in the second Group the absolute and relative additional impact of the nonavalent *vs* quadrivalent vaccine was 18.2% and 35.7%, respectively, Tables 2 and 4.

With concern to 297 partners of positive females (Group III), the potential impact of the nonavalent vaccine against quadrivalent vaccine was effective for both the low impact 26.3% > 15.1% (p < 0.0001) and the high impact 33.7% > 23.2% (p < 0.0001). The benefit of the nonavalent vaccine compared to quadrivalent vaccine, as shown by the absolute and relative additional potential impact, was 21.5% and 56.1%, respectively.

## Discussion

The current study provides an important overview on HPV-DNA prevalence in men with and without clinical manifestations and on the evaluation of the potential impact that nonavalent HPV vaccine has on HPV infection compared to the previously used quadrivalent HPV vaccine. The 58.7% of the samples tested were positive for HPV, with significant differences depending by considered group. In fact, 69% of men with clinical symptoms, 44.3% of those with a risky sexual contact (STD), and 63.7% of men with positive partners, were positive results to the HPV infection. HPV infection is very common in men, with variations reflecting the selection criteria of the studied populations and sampling methods used^14,32^.

The prevailing goal is to try to understand the relationship between HPV infection and disease in men, including the development of genital warts, penile intraepithelial neoplasia and invasive penile carcinomas. Genital warts are caused by HPV6,11 and are the most common clinical manifestation of HPV in men. Although they are benign and not associated with mortality, they are a source of psychosocial and physical discomfort^33^.

From the statistical analysis of the data it is highlighted that in group II, of subjects with sexual contacts at risk, a higher age was observed, suggesting once again to extend the vaccination also to adult men. This vaccination policy, in Sicily, is also applied to women. The vaccine is free for women up to 45 years of age.

Variations emerged also regarding the most frequent genotypes in the three groups, confirming how the selection of the target population is important in epidemiological assessments.

In our study, multiple HPV-type infections were shown in 51.2% without significant differences between the groups. Little is known about the prevalence of multiple infection HPV and associated factors in men. In women, two cohort studies showed contradictory results; one did not find any association between the number of HPV types and the cytological changes shown^34^, while the another one found strong associations between high grade squamous intraepithelial lesions and the number of HPV-types^35^. Furthermore, in a case–control study, women with cervical intraepithelial neoplasia 1 or worse were more likely to have more HPV types than women with normal cytology^36^. The role of multiple HPV infection in male health is not yet clear^37^.

Infections with multiple genotypes seem to favour the persistence of HPV^38^ and have been associated with a longer duration of infection^39^. In general, HPV infection is complicated by the existence of multiple infections; therefore, the potential benefit of the HPV vaccine is not easily assessable.

Oncogenic genotypes have been found in almost 80% of the men included in our study. The prevalence of hrHPV in the three different groups was statistically significant. In 84.5% of partner men of HPV-positive women, hrHPV was shown, suggesting a possible role of man in the transmission of the infection. Furthermore, high and significant prevalence was observed also in men with risky intercourse, again suggesting the need to make vaccination offerings as gender-neutral as possible.

We should also note that no screening tests are currently available for men, and that persistent penile HPV infections have been shown to be associated with penile intraepithelial neoplasia (PIN) development in younger men. These generally resolve within 2 years; however, a small minority of cases can progress and cause invasive cancer. Penile cancer in the United States has an incidence rate of around 1/100,000, while the incidence in some developing countries, such as Uganda, can be much higher (4.4/100,000)^40^. This occurs in these countries due to the immunocompromised caused by HIV infection^41^. In contrast, in women a decrease in dysplasia has been observed thanks to screening programs but overall thanks to vaccination^40^.

Impact of the vaccine was measured according to low and high impact parameters and defined in statistical analysis section. These parameters were similar to parameters adopted by Riethmuller et al.^24^, but we consider positive HPV patients only, to evaluate the effectiveness of the two vaccine.

On total HPV positive sample we observed that nonavalent vaccine had a greater impact than the quadrivalent vaccine. In fact, the absolute additional impact of the nonavalent vaccine compared to the quadrivalent was of the 20.1%, while the relative additional potential impact was 47.2% (Table1). The estimated coverage for the nonavalent vaccine was also significant within the groups considered. In fact, a significant relationship between AAI and RAI with groups was observed (p = 0.0157, < 0.0001 respectively, Table 2). The same result is described in a study on the women with high- and low-grade lesions. In this case, the additional impact of the nonavalent vaccine compared to the quadrivalent vaccine was over 80% for low-grade and over 50% for high-grade lesions^25^.

The approach used, shows clearly that the quadrivalent vaccine is more suitable for men with genital lesions in comparison to other groups (58%, p = 0.0153), while the nonavalent is suitable for all three groups, confirming that the transition from quadrivalent to nonavalent is an excellent prevention strategy (Table 3). In fact, the nonavalent vaccine showed no significant differences among groups about percentage of patients with genotype included in nonavalent vaccine (p = 0.068).

In this study, the effectiveness of the vaccine was calculated with low and high impact parameters. In particular, low impact was calculated considering HPV genotypes alone or in association, by excluding the presence of any other HPV type; while high impact was calculated considering the vaccine genotypes associated with other genotypes. This configures the risk of an overestimation of the vaccine's degree of effectiveness. Therefore, it can be reasonably assumed that the potential real impact of the vaccine is in the middle between high and low impact^24^.

In men in this study, both the low and high impact of the nonavalent vaccine were significant. In particular, the low impact for the nonavalent vaccine was significantly higher than the quadrivalent vaccine, for men with lesions and risky sexual behaviours, (p = 0.0078, p < 0.0001 respectively). This is probably due to the fact that the nonavalent vaccine contains, in addition to HPV16 and HPV18, five other high-risk genotypes, which together are responsible for about 90% of cancers worldwide^42^.

As for the partners of positive women, the nonavalent vaccine had a significant impact for both the low and high impact (both p < 0.0001, Table 4). The association with genotypes other than those contained in the vaccine plays a decisive role in the prevention determined by vaccination with the nonavalent versus the tetravalent.

Several countries recommend gender-neutral vaccination indeed. The infection supported by HPV has a great impact also on men. In Europe, 14,700 annual cases of anogenital tumours are attributable to HPV, with 5400 cases diagnosed in men (about half in the anus and half in the penis). With regard to precancerous lesions, it is estimated that over 1000 cases of AIN2/3 are diagnosed in men each year^43^.

Head and neck cancers also constitute a heavy burden, particularly in men, with an estimated 11,000 cases diagnosed annually. Further, increasing trends in the incidence of HPV-positive head and neck cancers have been consistently observed in the last decade in concomitance with the decline in tobacco use^44^.

There is evidence to suggest that where there is a high level of vaccination coverage in women, males are indirectly protected from infection. In Australian heterosexual men under the age of 25, the prevalence of HPV16,18, 6,11 decreased by 78% from the pre-vaccination period^45^.

In the context of female-only vaccination, the indirect advantage of herd protection among men who have sex with men are limited^46^. For reasons of fairness, some believe it is preferable for both males and females to have access to the same vaccination opportunities^47^. In conclusion, the present study on a male population in Sicily (Southern Italy) confirms that the transition from quadrivalent to nonavalent HPV vaccine offers much wider protection even in men, although with some differences between the analysed groups. The nonavalent vaccine respect to quadrivalent is to be preferred especially in men with positive HPV partners, who would have a potential role in the transmission of the infection, and in the eventual relapse after treatment. However, it should also be emphasized, that a significant reduction in HPV related infections, both in men and women, can be achieved only if the vaccination coverage reaches and exceeds 80%^48^.

Adequate vaccination schedules could therefore become an economically viable approach to prevent public health^49^.

The implementation of vaccination programs and gender-neutral vaccination represent the main way to go in the prevention of HPV related diseases in both men and women.
